# Beekeeping Management Practices Are Associated with Operation Size and Beekeepers’ Philosophy towards in-Hive Chemicals

**DOI:** 10.3390/insects10010010

**Published:** 2019-01-08

**Authors:** Robyn M. Underwood, Brenna E. Traver, Margarita M. López-Uribe

**Affiliations:** 1 Department of Entomology, Pennsylvania State University, 501 ASI Building, University Park, PA 16802, USA; 2 Department of Biology, Penn State Schuylkill, 200 University Drive, Schuylkill Haven, PA 17972, USA; bet12@psu.edu; 3 Department of Entomology, Pennsylvania State University, Center for Pollinator Research, University Park, PA 16802, USA; mml64@psu.edu

**Keywords:** honey bee, beekeeper, management, organic, conventional, treatment free

## Abstract

Management by beekeepers is of utmost importance for the health and survival of honey bee colonies. Beekeeping management practices vary from low to high intervention regarding the use of chemicals, hive manipulations, and supplemental feeding of colonies. In this study, we use quantitative data from the Bee Informed Partnership’s national survey to investigate drivers of management practices among beekeepers in the United States. This is the first study to quantitatively examine these variables to objectively describe the management practices among different groups of beekeepers in the United States. We hypothesized that management practices and goals among beekeepers are different based on the beekeeper’s philosophy (as determined by their willingness to use chemicals to control pests and pathogens) and the size of the beekeeping operation. Using a multiple factor analysis, we determined that beekeepers use a continuum of management practices. However, we found that beekeepers’ willingness to use in-hive chemicals and the number of colonies in their operation are non-randomly associated with other aspects of beekeeping management practices. Specifically, the size of the beekeeping operation was associated with beekeepers’ choices of in-hive chemicals, while beekeepers’ philosophy was most strongly associated with choices of in-hive chemicals and beekeeping goals. Our results will facilitate the development of decision-making tools for beekeepers to choose management practices that are appropriate for the size of their operations and their beekeeping philosophy.

## 1. Introduction

**Figure 1:**
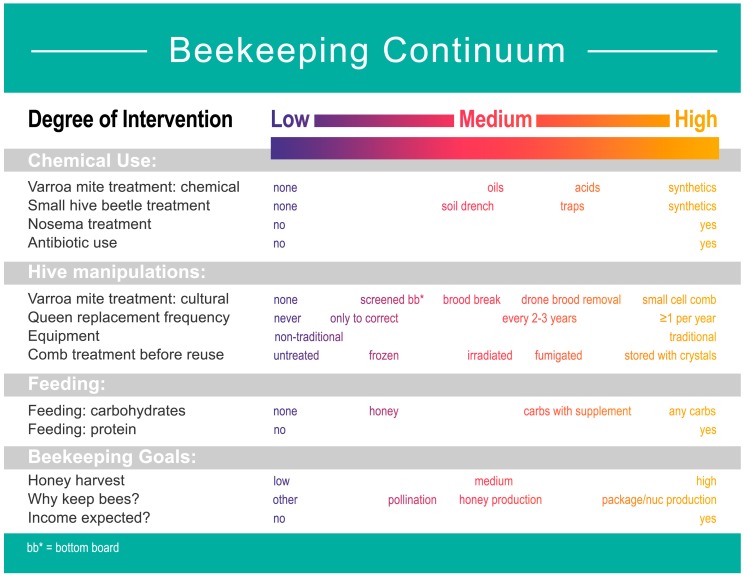
The continuum of beekeeping choices. Beekeepers can choose from a variety of management practices for chemical use, hive manipulations, feeding, and beekeeping goals. The degree of intervention is higher when (1) a chemical is present, as the raw product, or as a residual contaminant, for a longer time, (2) the honey bee colony is more fundamentally manipulated, or (3) the product requires more bee manipulation prior to use. Image by Nick Sloff.

Honey bees, *Apis mellifera* L., are the most important managed pollinators for agriculture, providing direct benefits to crops valued at $5–10 billion annually in the United States [1]. However, the annual loss of managed colonies has significantly increased in North America and Europe for a variety of reasons, including reduction of available floral resources, increased use of pesticides, and emerging diseases [2,3,4]. Beekeeping management practices are considered key for the productivity, overall health, and overwintering success of managed honey bee colonies [5]. Control of a number of parasites and pathogens is a priority, because they are among the main causes of summer and winter colony losses [6]. When making choices concerning honey bee management, a continuum of options is available (Figure 1). However, it is unclear whether beekeepers’ choices along this continuum are random or if certain combinations of choices are associated with each other (e.g., high vs. low intervention options). 

Out of all the pests and diseases that honey bees face, the ectoparasite *Varroa destructor* (commonly known as the varroa mite) is the most devastating, because it weakens colonies by vectoring several viruses [7]. Varroa mite control is currently considered one of the most important practices for successful beekeeping [7]. There are a variety of legal, registered chemical control options available to beekeepers, ranging from synthetic chemicals (e.g., amitraz and *tau*-fluvalinate) to organic compounds (e.g., formic and oxalic acids and essential oils), all of which vary in how they are applied, their mode of action, and their efficacy (see review by [8]). Alternatively, cultural non-chemical control methods could be used while using an integrated pest management (IPM) approach, which does not rely on in-hive chemicals as the first option for mite control. Cultural IPM approaches for varroa mite control include hive manipulations (e.g., small cell combs, mite trapping in drone brood, screened bottom boards) and interruption of the brood cycle. These IPM approaches can be used alone or in combination with chemical controls.

Other hive pests, including insects, microsporidia, and bacteria, also require management in beekeeping operations. These include small hive beetles, nosema disease, and foulbrood. Management of these pathogens can include chemical treatments and hive manipulations. Small hive beetles (SHB), *Aethina tumida*, are an invasive pest of honey bee colonies that destroy combs, defecate in the honey causing fermentation [9,10,11,12], and can vector both bacterial and viral pathogens [13,14,15]. Control options for SHB include the use of in-hive synthetic chemicals (e.g., coumaphos), drenching the soil with chemicals (e.g., permethrin) or natural enemies (e.g., nematodes), and using physical traps with lures. *Nosema apis* and *N. ceranae*, collectively known as nosema, are microsporidian gut parasites that can cause increased colony mortality, immune suppression, and earlier foraging in honey bees (reviewed in [16,17,18]). The most common treatment that is used for nosema infections is fumagillin, sold as Fumagilin-B. However, the supplier, Medivet Pharmaceuticals, discontinued producing the chemical inventory as of April 2018 [19]. Old inventory is currently the only available source. Natural compounds (e.g., thymol, Nozevit, and resveratrol) and non-chemical control strategies can also be employed to target the nosema spores [20,21,22,23]. Non-chemical practices to control nosema spores on equipment include heat treatments, freezing, and gamma irradiation [24,25,26]. American and European Foulbrood (*Paenibacillus larvae* and *Melissococcus plutonius*, respectively) are bacterial infections that, if left untreated, can lead to high levels of brood mortality. The most effective option for treatment is to burn the bees and equipment, but the bees can also be shaken onto new equipment and treated with antibiotics (e.g., terramycin). However, recent laws in the United States require a prescription from a veterinarian [27] to purchase antibiotics for these treatments, effectively making their use cost prohibitive and logistically difficult.

Additional beekeeping management choices along the continuum involve the supplemental feeding of carbohydrates and/or protein and the type of equipment used for beekeeping. Carbohydrate feeds, such as sucrose syrup (with or without essential oils or other supplements) or high fructose corn syrup, are commonly fed in the spring to simulate a nectar flow and encourage brood rearing. These supplemental feedings are also used in the fall to encourage storage for winter [28]. Most beekeepers are generally willing to supplement colonies by feeding carbohydrates to prevent starvation, but there is variability in the amount and type of feed provided. Protein feeds, known as pollen substitutes, are an inexpensive way for beekeepers to increase the protein that is available to honey bees and encourage population build-up during times of limited pollen forage in the landscape [29,30]. However, the addition of protein is not a universal practice among beekeepers, nor is it necessary for colony survival. Finally, the choice of hive equipment also varies among beekeepers, but one common theme is that they all contain removable frames, as required by most states [31]. The traditional Langstroth-style equipment is the most common [32], but non-traditional options, such as top-bar and warre hive, are also used [33]. Most beekeepers use frames with plastic or wax foundation (embossed hexagons), but some use foundationless frames. Beekeepers that prefer to use foundation can choose from several cell sizes, with hexagons varying from 4.9–5.4 mm in size. 

Among beekeepers, there is also a continuum in their beekeeping goals. Some beekeepers aim to sell bees, so they manage colonies for a large honey bee population early in the season to ensure the availability of large numbers of workers and queens in the spring. Other beekeepers aim to sell honey and they will maximize the worker population in each colony to match the timing of maximum forage. Still others aim to have many small colonies to rent out for pollination services. Hobbyist beekeepers, with small numbers of colonies and no expectation of income, will be highly variable in their management, keeping colonies for enjoyment, environmental stewardship, or for production of small amounts of honey.

In summary, beekeepers have a wide range of available options for controlling pests and pathogens, for feeding carbohydrates and protein, for housing honey bee colonies, and have different goals for their operations. These options fall on a beekeeping continuum, with each management decision having a range of possible choices (Figure 1). We hypothesize that the management practices and goals among beekeepers are non-randomly associated based on two criteria. Specifically, we hypothesize that practices and goals among beekeepers will be different based on (1) the beekeeper’s philosophy (determined by their willingness to use chemicals to control pests and pathogens (referred as beekeeping philosophy hereafter)) and (2) the size (number of colonies) of the beekeeping operation. To test these hypotheses, we used the national survey data collected by the Bee Informed Partnership (BIP). The BIP survey included specific questions that are related to various management choices, such as type of in-hive chemicals used, type of feeding and equipment used, goals of the beekeeping operation, operation size, and willingness to use in-hive chemicals. We used a multivariate approach to investigate the association between different management practices and beekeeping goals among beekeepers. We then used the calculated distance between beekeepers to investigate the presence of distinct groups of individuals that can be identified based on our two hypothesized factors. Our results support the presence of a continuum of management practices and goals among beekeepers. However, we also found that non-random associations in management practices and goals based on beekeeping philosophy and the size of their operations. In this article, we discuss, in detail, the differences and similarities among these groups and the implications of our results for how we understand the choices made by beekeepers when managing colonies.

## 2. Methods

We purchased raw data from the national honey bee colony management survey conducted by the Bee Informed Partnership from beekeepers reporting on the 2016–2017 beekeeping year (available at https://beeinformed.org/wp-content/uploads/2017/03/2016-2017-Loss-and-Management-Survey-PREVIEWfinal.pdf (accessed on 8 January 2018)). The survey contained 94 questions that encompassed all aspects of honey bee management. We used the 13 questions that are relevant to the variables of interest, which include type of in-hive chemicals used, type of feeding and equipment used, goals of the beekeeping operation, operation size, and willingness to use in-hive chemicals (Table 1). The data were first curated to eliminate all respondents that failed to answer one of the key questions included in our analyses. In cases where a respondent gave multiple answers to one of the questions about management practices, we used the most intensive practice in our data analysis (Figure 1). This manipulation was based on the assumption that beekeepers willing to use more intensive practices would be willing to use less intensive options, but not the other way around. To characterize a respondent’s beekeeping philosophy, we used the following question: “When choosing treatment or feeding strategies for your colonies, would you say that you…” (1) are not willing to use non-bee produced products in the hive, (2) are only willing to use natural or organic products in the hive, or (3a) prefer to use natural or organic products in the hive but will use synthetic products if needed, (3b) will use synthetic products if needed, or (3c) have no preference. Thus, we coded these groups as natural (1), organic (2), or conventional beekeepers (3a–c). The operation size was described using the question: “How many living colonies did you have on October 1, 2016”. Beekeepers were categorized as backyard if they had less than 50 colonies, sideline if they had between 50 and 499 colonies, and commercial if they had more than 500 colonies. Other aspects of management were characterized using the remaining set of questions, which were grouped into the following categories: chemical use, hive manipulations, feeding, and beekeeping goals (Table 1).

We used a multiple factor analysis (MFA) to investigate the association between different beekeeping management practices and goals and two supplementary variables: beekeeping philosophy (conventional, organic, natural) and operation size (commercial, sideline, backyard). This type of analysis is often used to analyze datasets where multiple variables are characterized for the same objects, but there is no a priori relationship of cause and effect among the variables (e.g., [34,35]). MFA is similar to a principal component analysis (PCA) with two main differences: (1) it is suitable for the analysis of categorical variables and (2) subsets of variables can be analyzed using weighted covariate matrices that homogenize variation between groups. MFA is comprised of two basic steps. First, each subset of variables is subjected to a PCA analysis and normalized by the first PCA eigenvalue. Second, these normalized subsets are merged into one matrix of values and individuals are projected onto a final global PCA using unique merged values. The global analysis weighs variables to balance the influence that each set of variables has on the results. To assess the degree of association between the different categories of beekeeper management practices and goals, we used the RV coefficients that are a multivariate generalization of the Pearson correlation coefficient. The interpretation of MFA plots of individuals and groups of variables is similar to PCA. Plots of superimposed individuals show the distance between these objects in the global multivariate space incorporating information from all variables. The directionality of the arrows in the partial axes plots of groups of variables indicate how the effects of these variables are correlated with each other, while the length of the arrow indicates the effect size. The test value (v.test) indicates whether the categories of the supplementary variables (beekeeping philosophy and operation size) are significantly different from zero, which for our dataset is the null of the composite of beekeeping management practices and goals. All of the analyses were performed in RStudio v. 1.1.447 in the package FactoMineR v.1.39 [36,37]. 

## 3. Results

**Figure 2:**
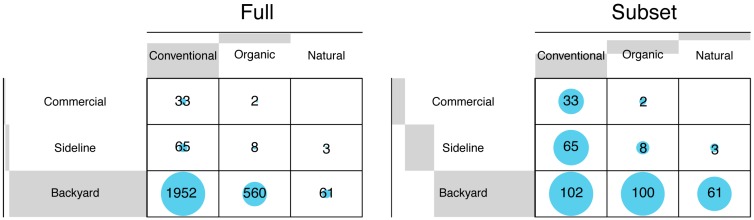
A comparison of the number of responses in full dataset with the subset of data that were used for analysis. There were no commercial beekeepers with a natural beekeeping philosophy.

A total of 4963 beekeepers in the United States, representing 363,987 honey bee colonies, participated in the 2016–2017 BIP survey [4]. The curated database of usable beekeeping responses resulted in responses from 2684 participants. However, participants were not equally distributed across groups in the supplementary variables (Figure 2). Conventional beekeepers that were willing to use any chemical product and those with backyard operations accounted for 76% and 96% of the participants, respectively. We, therefore, used a randomly selected subset of the dataset for analysis and visualization, which only included 374 participants where no single group was represented by more than 30% of the participants. We performed the data analysis on the subset, and the results are comparable to the output of the analysis of the full dataset (Table S1).

We performed the MFA on six groups of variables (management philosophy, operation size, type of chemicals to control pest and diseases, type of supplemental feeding, type of hive manipulation, beekeeping goals), while two variables (management philosophy and operation size) were not used for the construction of the global PCA of the MFA (supplementary variables). Cumulative variance associated with the first 10 MFA eigenvalues was 61%, with the first two accounting for 21.3% of the variation (Table S2). The MFA results indicated that beekeepers could be separated into categories based on their beekeeping philosophy and the size of their operation supported by the v.test that was significantly different than zero for all groups, except for natural beekeepers on dimension 2 and backyard beekeepers on dimension 1 (Table 2). 

**Figure 3:**
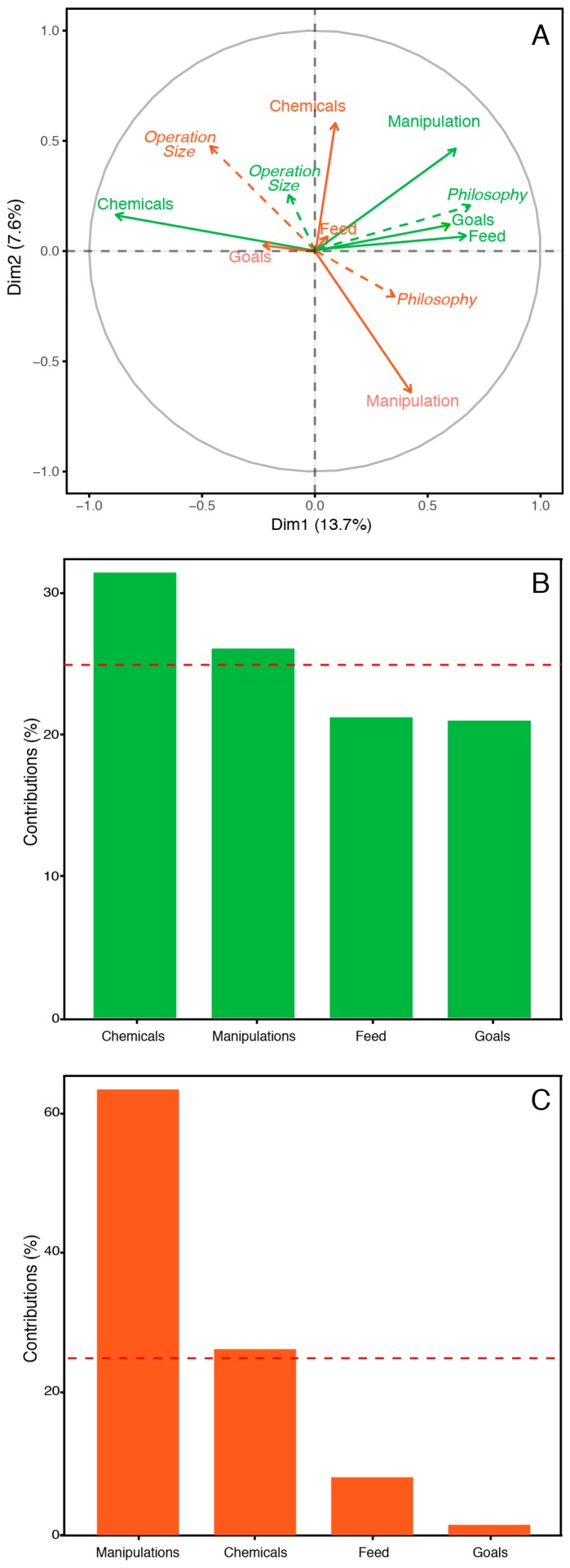
Results of variables reduced through the Multiple Factor Analysis (MFA). (**A**) Relationship between supplementary variables (beekeeping philosophy and operation size; dotted lines) and other management practices (chemical use, feeding, hive manipulations and goals; solid lines) summarized in partial axes onto MFA dimensions 1 (green) and 2 (orange). Practices based on beekeeping philosophy are strongly correlated with type of hive manipulation, type of feed and beekeeping goals. Practices based on operation size are correlated with the type of in-hive chemicals used. (**B**) Contribution of the different groups of variables to the variation captured in MFA dimension 1. (**C**) Contribution of the different groups of variables to the variation captured in MFA dimension 2. Horizontal dotted lines (**B**,**C**) indicate the threshold of variables that contribute to more than 25% of the variation on each axis.

The first two dimensions of the MFA grouped different aspects of the variation among beekeepers (Figure 3). All of the variables showed high loadings in the first axis, while beekeeping goals and supplementary feeding had low loadings on dimension 2 (Figure 3A). Dimension 1 captured the highest contribution of variation from in-hive chemicals and hive manipulations (Figure 3B), and dimension 2 captured most of the variation from hive manipulations (Figure 3C). Association between groups of variables show that management philosophy was most correlated with in-hive chemicals (RV = 0.26) and beekeeping goals (RV = 0.27). Operation size was most strongly correlated with in-hive chemicals (RV = 0.18) (Table S3).

**Figure 4:**
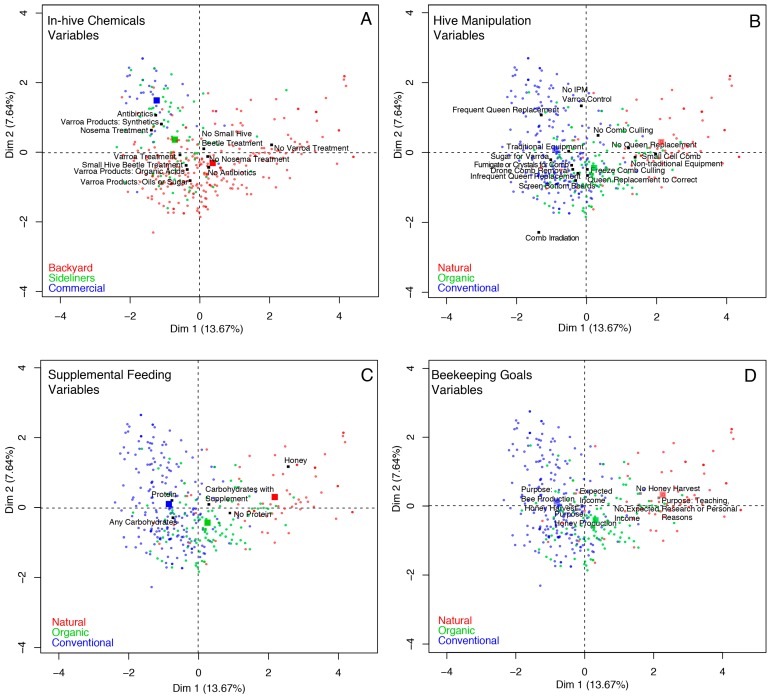
Scatterplots summarizing the distance among 374 surveyed beekeepers onto the first two axes of MFA. Survey participants are colored by their operation size (**A**) and their beekeeping philosophy (**B**–**D**). Large colored squares indicate the centroid of each group. Small black squares indicate the position of the centroid of each categorical variable.

The distance between individuals in MFA space indicated the presence of a continuum among beekeepers while supporting the presence of different groups based on operation size (Figure 4A) and beekeeping philosophy (Figure 4B–D). The data show that the greatest variability is present among conventional beekeepers (Figure 4B–D) and among backyard beekeepers (Figure 4A). Despite the presence of this variability, the data support differentiation in the management practices and goals of beekeepers, particularly for two groups: natural and commercial beekeepers. Natural beekeepers, defined as those not willing to use chemicals in their hives, clearly use different practices and have different goals for their beekeeping operations. The most unique characteristics of natural beekeepers are that they tend to use small cell combs in their frames, use non-traditional equipment, feed colonies honey, do not treat colonies for varroa mites, and use the colonies for teaching, research, or other personal reasons (Figure 4B–D). On the other hand, commercial and sideline beekeepers, defined as those that have more than 50 colonies, are uniquely characterized by frequent queen replacement, the lack of use of integrated pest management (IPM) practices or equipment to control varroa mites, the use of antibiotics and synthetic chemicals, and the use of protein and carbohydrate supplemental feedings (Figure 4A).

## 4. Discussion

Survey data showed that beekeepers use a continuum of beekeeping practices and goals, as predicted (Figure 1). However, we found evidence that subsets of beekeepers’ management practices and goals are associated with beekeeping philosophy and operation size (Figure 3 and Figure 4). When management practices and goals were categorized by beekeeping philosophy, we found general groups of practices that differentiate conventional, organic, and natural beekeepers. In general, conventional beekeepers tend to vary widely in their practices, but they use frequent queen replacement, antibiotics, and synthetic chemicals for pest and pathogen control, supplement colonies with protein and carbohydrates, and expect an income from their operations (Figure 4B–D). Organic beekeepers tend to use alternative methods for pest control, which include freezing comb prior to reuse, screen bottom boards, and drone brood removal (Figure 4B–D). Organic beekeepers also tend to replace queens less frequently than beekeepers in the conventional group. The tendency for natural beekeepers is to use no treatment for varroa mites, use small cell comb, no queen replacement, non-traditional equipment, and feed colonies with honey or syrups that contain a supplement (Figure 4B–D). Most natural beekeepers do not have financially driven operations through bee or honey production, but keep bees for enjoyment, teaching, or research, and they are less likely to earn an income from their operations (Figure 3C). 

When management practices and goals were categorized by operation size, we also found general groups of practices that differentiated beekeepers with commercial, sideline, and backyard operations. Beekeeping management by commercial and sideline beekeepers is largely different from practices of backyard beekeepers (Figure 3A). Commercial and sideline beekeepers more consistently use antibiotics and synthetic chemical treatments to control pests and parasites than backyard beekeepers (Figure 3A). On the contrary, backyard beekeepers vary more widely in their choices of in-hive chemicals, sometimes using organic acids or essential oils, and sometimes avoiding the use of chemicals altogether. Other practices, such as frequent queen replacement and not using IPM practices or equipment for varroa mite control, are more common among commercial and sideline beekeepers. For supplemental feeding, commercial and sideline beekeepers use pollen and carbohydrate supplements, while backyard beekeepers range from the use of both to not using protein to the exclusive use of honey for carbohydrate supplementation. The BIP survey captured few natural and organic beekeepers with more than 50 colonies represented in this survey (Figure 2). The low representation of organic and natural beekeepers with large operations could be the result of uneven sampling effort to survey these groups of beekeepers. Alternatively, it may indicate barriers to increasing colony numbers when using organic or natural beekeeping management practices.

Overall, our findings suggest that beekeepers most likely make management decisions by weighing the financial and biological benefits of each practice. For example, because commercial and sideline beekeepers regularly use chemical treatments to keep varroa mite levels below two mites per 100 bees, they require the use of strong chemical treatments that kill 60–95% of the mite population [8,38]. On the other hand, backyard beekeepers choose to use a wider range of options that include more labor-intensive practices, such as drone brood removal. The financial costs of highly effective chemical treatments are probably counterbalanced by the decrease in required per-hive labor to apply these chemicals on a large scale and a decrease in winter mortality. According to the data that are freely available on the beeinformed.org website, winter mortality differed by operation size; backyard beekeepers lost an average of 45.5% of their colonies over the winter of 2016–2017, while sideliners lost 37.2%, and commercial beekeepers lost only 25.6%. These results about winter mortality indicate that colony survival is affected by management practices.

The Bee Informed Partnership’s Management Survey provided an invaluable source of data to examine the management practices of beekeepers using a quantitative approach. This comprehensive set of questions was used to elucidate how management practices and goals vary among beekeepers that have different management philosophies and operation sizes. However, surveys have some limitations, as questions can be interpreted by respondents differently. For example, one survey question asked “Did you use any of the following IPM practices/equipment to try to control varroa mites?”. One possible answer was “powder sugar.” The intent of this question was to determine if beekeepers were using a powdered sugar treatment for control of varroa mites [39]. To our knowledge, this is not a widely used practice among conventional beekeepers, because it is time consuming. The appearance of this management practice as something that is used by conventional beekeepers (Figure 2A) suggests that respondents thought of this option as a sugar roll, which is not a treatment, but a means for monitoring for varroa mite levels [40]. Also, keeping bees with the goal of commercial pollination of crops was not considered as one of the possible goals of beekeeping operations in this study, but 11% of beekeepers nationwide fill this niche (https://bip2.beeinformed.org/survey/ (accessed on 18 August 2018)). Management practices among these beekeepers could be different from those that were analyzed here. These types of limitations that are associated with survey data must be considered when interpreting results. 

## 5. Conclusions

Our study provides the first empirical assessment of the associations between management practices and goals among beekeepers, and sheds light on the potential drivers of different sets of management choices. We describe, for the first time, the existence of three groups of beekeepers based on their philosophy towards in-hive chemicals (conventional, organic, and natural) and characterize, in detail, common practices that are used by each of these groups. Differences in the management practices between commercial, sideline, and backyard beekeepers are often mentioned in the literature, but had not been previously characterized (e.g., [4,41,42]). Because there is a continuum of beekeeping practices, most beekeepers do not necessarily fall into one discrete category. However, we show that beekeeping management practices are not randomly selected among individuals. How this continuum of management practices impacts colony health, productivity, and overwintering success remains to be investigated. Future work should aim to determine the biological and economic effects of these management decisions. 

## Figures and Tables

**Table 1:** Questions from the Bee Informed Partnership 2017 beekeeping management survey used to analyze associations between beekeeper management practices in the United States.

Factor	Survey Question ^1^	Response Category ^2^
Chemical Use	Which, if any, of the following did you apply to any of your colonies between April 1, 2016 and April 1 2017?	
	Varroa product used	Synthetics
		Organic acids
		Essential Oils
		None
	Nosema product used	Y/N
	Antibiotics used	Y/N
	Small hive beetle treatment	Y/N
Feeding	Which, if any, of the following did you apply to any of your colonies between April 1, 2016 and April 1 2017?	
	Carbohydrates	Any carbs
		Carbs with supplement
		Honey
		None
	Protein	Y/N
Hive manipulations	Did you use any of the following IPM practices/equipment to try to control varroa mites?	
		Small cell comb
		Drone brood removal
		Screened bottomboards
		Brood break
		None
	Generally, how often do you replace queens in your colonies?	
		Frequently
		Infrequently
		To correct
		Never
	What hive type did you use to keep your colonies?	
		Traditional
		Non-traditional
	Last year, what did you do before you re-used brood comb that you have taken out of production or purchased?	
		Crystals or fumigate
		Irradiated
		Froze
		Nothing
Beekeeping goals	Why did you keep bees?	
		Bee production for sale
		Honey production for sale
		Other
	Were you expecting to derive an income from your beekeeping activities?	Y/N

^1^ Question from the Bee Informed Partnership (BIP) 2017 survey. Full questions can be found at https://beeinformed.org/wp-content/uploads/2017/03/2016-2017-Loss-and-Management-Survey-PREVIEWfinal.pdf; ^2^ Coded responses beekeepers provided for the survey.

**Table 2:** Numerical results of the multiple factor analysis for the supplementary variables. Values indicate the effect size of each variable for dimensions 1 and 2. Values greater than 2 for the v.test indicate significant differences between the centroid of each group and zero.

		Dimension 1	v.test	Dimension 2	v.test
Beekeeping Philosophy	Natural	82.887	19.313	0	0
	Organic	6.072	−5.664	64.516	18.464
	Conventional	11.040	−9.408	35.484	−16.867
Operation Size	Backyard	0	0	29.679	−19.313
	Sideline	31.531	−12.149	48.148	15.013
	Commercial	68.468	16.786	22.173	9.552

## Supplementary Materials

The following are available online at http://www.mdpi.com/2075-4450/10/1/10/s1, Table S1: Numerical results of the multiple factor analysis for the supplementary variables of the full dataset. Values indicate the effect size of each variable for dimensions 1 and 2. Values greater than 2 for the v.test indicate significant differences between the centroid of each group and zero. This table is comparable to Table 2 in the manuscript, Table S2: Summary of the first 10 eigenvalues of the multiple factor analysis (MFA), Table S3: Pairwise RV coefficients between groups of variables used in the multiple factor analysis (MFA).
