# Supplementary material for: Beekeeping Management Practices Are Associated with Operation Size and Beekeepers’ Philosophy towards in-Hive Chemicals
Source: Insects. 2019 Jan 8;10(1):10. doi: 10.3390/insects10010010 (PMC6359672; doi:10.3390/insects10010010)
Supplement: Supplementary file 1 [file insects-10-00010-s001.pdf]

Table S1. Numerical results of the multiple factor analysis for the supplementary variables of the full dataset. Values indicate the effect size of each variable for dimensions 1 and 2. Values greater than 2 for the v.test indicate significant differences between the centroid of each group and zero. This table is comparable to Table 2 in the manuscript.

		Dimension 1	v.test	Dimension 2	v.test
Beekeeping Philosophy	Natural	-1.194	-7.317	0.557	4.299
	Organic	-0.531	10.806	-0.006	-0.144
	Conventional	0.185	13.033	-0.016	-1.405
Operation Size	Backyard	-0.080	15.128	-0.087	-20.746
	Sideliners	1.733	11.603	1.636	13.785
	Commercial	2.126	9.587	2.865	16.256

Table S2. Summary of the first 10 eigenvalues of the multiple factor analysis (MFA) of the subset of data.

	Dim.1	Dim.2	Dim.3	Dim.4	Dim.5	Dim.6	Dim.7	Dim.8	Dim.9	Dim.10
Variance	2.14	1.20	0.97	0.91	0.86	0.80	0.75	0.71	0.68	0.65
% Variance	13.7	7.6	6.2	5.8	5.5	5.1	4.8	4.6	4.4	4.1
Cumulative	13.7	21.3	27.5	33.3	38.8	43.9	48.7	53.3	57.6	61.8

Table S3. Pairwise RV coefficients between groups of variables used in the multiple factor analysis (MFA) of the subset of data.

	Philosophy	Operation Size	Chemicals	Feeding	Manipulation	Goals	MFA
Philosophy	1.000	0.104	0.258	0.083	0.108	0.265	0.291
Operation Size	0.104	1.000	0.177	0.032	0.086	0.028	0.144
Chemicals	0.258	0.177	1.000	0.114	0.125	0.094	0.532
Feeding	0.083	0.032	0.114	1.000	0.041	0.036	0.464
Manipulation	0.108	0.086	0.125	0.041	1.000	0.071	0.728
Goals	0.265	0.028	0.094	0.036	0.071	1.000	0.446
MFA	0.291	0.144	0.532	0.464	0.728	0.446	1.000
